# Spinal Cavernomas: Outcome of Surgically Treated 10 Patients

**DOI:** 10.3389/fneur.2017.00672

**Published:** 2017-12-20

**Authors:** Ibrahim Sun, M. Necmettin Pamir

**Affiliations:** ^1^Neurosurgery, Acıbadem University, Istanbul, Turkey

**Keywords:** spinal cavernoma, spinal diseases, spinal surgery, cavernoma, cavernous angioma

## Abstract

**Aim:**

We report the preoperative and postoperative findings and also neurological follow-up results from 10 spinal cavernoma patients treated in our clinic. Several representative cases are presented in terms of clinical features, imaging results, and surgical outcomes.

**Material and methods:**

The data were retrospectively collected from patients’ files in the hospital records and sorted with regards to clinical presentation, radiologic features, and operative findings. Patients received spinal MRI scans for the diagnosis of spinal cavernomas (SC) and postsurgical evaluation. Clinical presentation was evaluated *via* Ogilvy classification and symptoms were checked preoperatively and postoperatively at third month and first year using McCormick scale. Primary treatment was microsurgical operation aiming a gross total lesion resection.

**Results:**

10 spinal cavernoma patients between the ages 30 and 63 were treated. Six (60%) of the patients were diagnosed with cervical and four (40%) others were diagnosed with thoracic SC. Among the patient group, mean preoperative Ogilvy classification score was 2.3 ± 0.7.8 and McCormick score was 1.9 ± 0.7. There was no residual mass or relapse after surgery. One patient developed surgery-related left hemiparesis, which was normalized at 1 year follow-up.

**Conclusion:**

Patients must be diagnosed with MRI since it is nowadays a gold standard. Preoperative and postoperative scores are important in evaluating the patients’ condition and improvement. The results from our patient series also reinforce the notion that immediate surgery should be the preferred treatment method for cavernomas. Intraoperative neurophysiologic monitarization should assist the surgery in order to prevent complications. In conclusion, microsurgery is a gold standard method that we recommend for cases of cavernomas, which will not recur if gross total resection is achieved.

## Introduction

Cavernous angiomas or cavernomas are abnormal clusters of small sinusoidal type capillaries and venules that create a lesion in the central nervous system (CNS). They may be observed in any part of the CNS, but they are most common in the supratentorial cerebral compartment (1, 2). Estimations indicate that prevalence rates reach up to 0.13% (3). Spinal cavernomas (SC) are rare lesions with an overall incidence rate of 0.04–0.05% in populations (4). These vascular pathologies constitute 3–5% of all CNS lesions and account for approximately 15% of all spinal vascular malformations (5). Sporadic and familial forms and -albeit being rare- *de novo* developed cavernomas have been described in the literature (6). Familial disposition is seen up to 50% with an autosomal dominant inheritance and loss of CCM1 (also known as KRIT1) protein was found to be an important factor in the development of cavernomas (7, 8).

Spinal cavernomas are mainly diagnosed *via* neurological deficits and complaints of the patients, but they may be also diagnosed incidentally on rare occasions. Clinical representation depends on the location and growth rate of the lesion, but common features include spinal pain, radiculopathy, progressive paraparesis, acute paraplegia, and progressive myelopathy (2). SCs are angiographically occult lesions; therefore, MRI is, nowadays, the gold standard radiological modality for diagnosis.

Conservative treatment of SCs is risky due to the possibility of bleeding and compression, which may cause neurological deterioration. Microsurgery is the accepted treatment method, especially for lesions, which are greater than 5 mm and which are located posteriorly. No recurrence is reported in the literature after the achievement of a successful gross total resection.

In this paper, we present 10 cases with microsurgically treated symptomatic intramedullary SCs between the years of 2005 and 2016. We report the preoperative and postoperative findings along with neurological follow-up results from 10 spinal cavernoma patients treated in our clinic. All patients underwent microsurgical operation, aiming a gross total resection. The preoperative clinical finding was scored *via* the grading system proposed by Ogilvy (9). Observed symptoms were classified and graded preoperatively and postoperatively, at 3 and 12 months; using the McCormick classification.

## Materials and Methods

### Patients’ Characteristics

The patient series consisted of five male and five female patients with ages ranging from 30 to 63 years old with an average age of 40.5 (Table 1). There were no familial cases and no genetic testing for KRIT mutations was performed. The data were retrospectively collected from patients’ files from the hospital records and sorted with regards to clinical presentation, radiologic features, and operative findings. Clinical presentation was evaluated *via* Ogilvy classification and symptoms were checked preoperatively and then postoperatively and at the third month and first year using the McCormick grading system.

**Table 1:** General characteristics of the patient series.

Age, years	40.5 ± 11.1
Male, *n* (%)	5 (50%)
Ogilvy score (median)	2
McCormick (median/preoperative)	2
McCormick (median postoperative 3rd month)	2
McCormick (Median postoperative 1st year)	1

### Radiologic Examination

MRI is the gold standard radiological method for diagnosis and follow-up of SC. It’s known that SCs are angiographically occult lesions. They may appear extramedullary or intramedullary and sometimes at extradural space. The mass was usually well marginated and there was no bone involvement in our patients. The lesion was mostly heterogeneous and was hypointense on T1-weighted images and hyperintense on T2-weighted images, in comparison to the intervertebral disk. On post-contrast, fat suppressed, T1-weighted images, the lesion may display heterogenous or homogenous enhancement.

It would be an alternative method, to perform angiography for patients who have not a distinctive, radiologic diagnosis. Since cavernomas are occult lesions, they will not appear as pathologic in angiography, therefore the diagnosis of carvernoma will be supported.

### Outcome Scores

Reported patients were all diagnosed with MRI and cavernoma was determined as the cause of the symptoms and complaints. Six of the cavernomas (60%) were in the cervical region and four of them (40%) were observed at thoracic level (Table 2).

**Table 2:** General information about the 10 individual patients included in this study.

Patient number	Age (years)	Lesion location	Follow-up (months)	Complications post-op 3rd Month	Complications post-op 1st Year
1	35–39	C1	126	None	None
2	60–64	T7	112	None	None
3	45–49	C3	77	None	None
4	35–39	C2	75	None	None
5	35–39	T11	76	None	None
6	55–59	C4	24	None	None
7	30–34	C2	17	None	None
8	30–34	C1	15	Left hemiparesis	Left hemiparesis
9	35–39	T8	13	None	None
10	30–34	T6	14	None	None

We investigated neurological symptoms and outcome using Ogilvy and Carter (9) and McCormick classifications (10). Of the 10 patients in our series, one presented with acute onset of stepwise deterioration (Ogilvy score 1), 6 with slow progressing neurological deterioration (Ogilvy score 2), 2 with acute onset of neurological deterioration with a rapid decline (Ogilvy score 3), and 1 with acute onset of mild symptoms of neurological deterioration with gradual decline over weeks to months (Ogilvy score 4). Assessments according to McCormick classification revealed three patients with grade 1, five patients with grade 2, two patients with grade 3 deficits. None of the patients exhibited grade 4 deficits. Preoperative mean Ogilvy scores were 2.3 ± 0.78 and mean McCormick scores were 1.9 ± 0.7 (Table 3).

**Table 3:** Neurological symptom scores (Ogilvy score) and neurological outcome scores (McCormick scores) of individual patients.

Patient number	Ogilvy score	McCormick pre-op	McCormick post-op	McCormick 3rd Month	McCormick 1st Year
1	2	1	1	1	1
2	3	2	2	2	2
3	1	2	2	2	1
4	2	1	2	2	2
5	3	2	2	2	1
6	2	2	1	1	1
7	4	1	1	1	1
8	2	3	4	2	1
9	2	2	1	1	1
10	2	3	2	1	1

### Surgery

All patients had spinal MRI for the diagnosis of SCs. There is no conservatively evaluated patient with SCs in our clinic. All patients were positioned in prone and all patients were explored with posterior approach. Cavernoma level was checked by fluoroscopy before skin incision. Midline skin incision was performed and laminas were exposed after bilateral subperiosteal muscle dissection and retraction. Total laminectomy was performed one to two levels according to cavernoma size. Then, the dura was opened and cavernoma was exposed. In the cases where cavernoma is localized anterolaterally, the method of cutting dentate ligament would be a good choice, because then, it will be possible to lift and turn anterolateral cord and expose anterolateral portion with a suture. The aim of the surgery is gross total resection. All patients’ specimen was sent for pathology check. All patients in this series were operated with intraoperative monitorization using somatosensorial-evoked potentials (SSEP). SSEP is a technique being used at integrity detection of spine intraoperatively. It uses the principle that a stimulation from peripheral is transmitted to the somatosensory cortex. It gives an electrical stimulation to peripheral receptors and checks somatosensorial response. In case of the disruption of the spinal integrity, the response from the somatosensory is disordered. Watertight closure of the dura mater is one of the most important levels of surgery. The cavernoma removal was confirmed in all patients with postoperative MR images in the first 24 h. Follow-up of the patients was performed on the first 45th day after the surgery. Then, patients were controlled after 3 months of the surgery. Radiologic control was performed at the third month and first years after discharge from the hospital. The patients are scheduled for control, once a year for 5 years long.

### Pathology Examination

Standard hematoxylin and eosin staining is performed on the removed lesion to confirm the cavernoma diagnosis. Histopathological examinations of excised lesions classically demonstrate compact clusters of thin-walled to thick-walled vessels without intervening neuropil. Reactive gliosis with hemosiderin-laden macrophages is viewed at the periphery of the lesions.

### Ethical Consideration

The ethical approval has been taken from “Acibadem University Ethics Committee of Consideration of Medical Research.”

## Results

**Figure 1:**
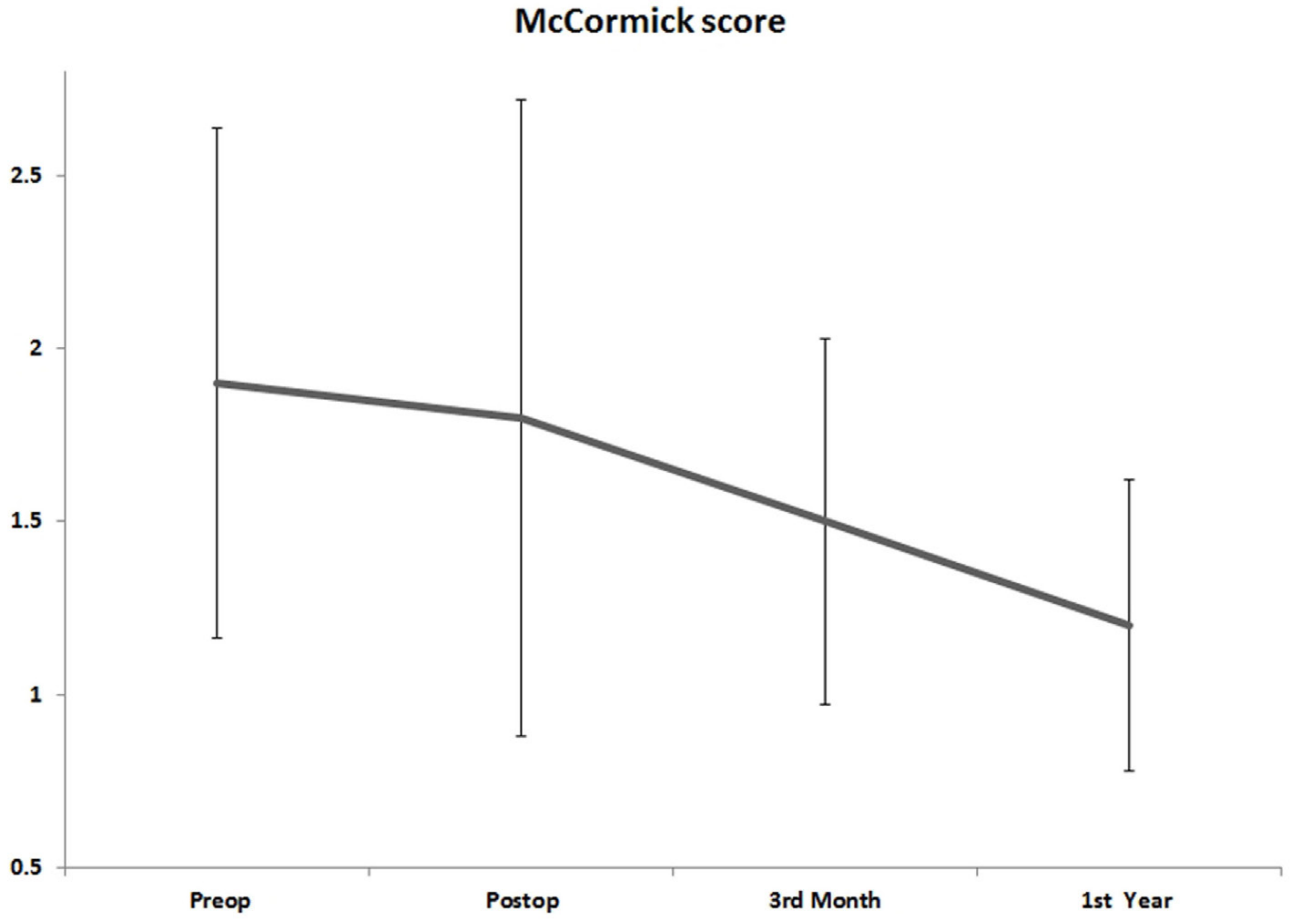
Mean McCormick scores of patients at preoperative and postoperative controls and follow-up controls at third month and first year.

No patients were treated conservatively. From the start, we do not recommend that. For all cases, after posterior midline incision and subsequent total laminectomy, cavernomas were excised and there is no case of mortality due to operations. Postoperative MRI revealed successful total resection without any residual mass or reoccurrence in all patients. At 1-year follow-up, McCormick scores were improved, eight patients were grade 1 and two patients were grade 2, respectively, with an average of 1.2 ± 0.4. Overall, eight patients showed neurological improvements, one remained in the same condition, and the condition of one patient slightly deteriorated, developing surgery-related left hemiparesis, which was normalized at 1-year follow-up (Table 3; Figure 1). Our study and findings indicate a favorable progress in neurological recovery after gross total resection and add more data for the below-given fact that surgical approach and total resection are considered to be beneficial when it comes to cavernomas.

### Representative Cases

#### Case-1

**Figure 2:**
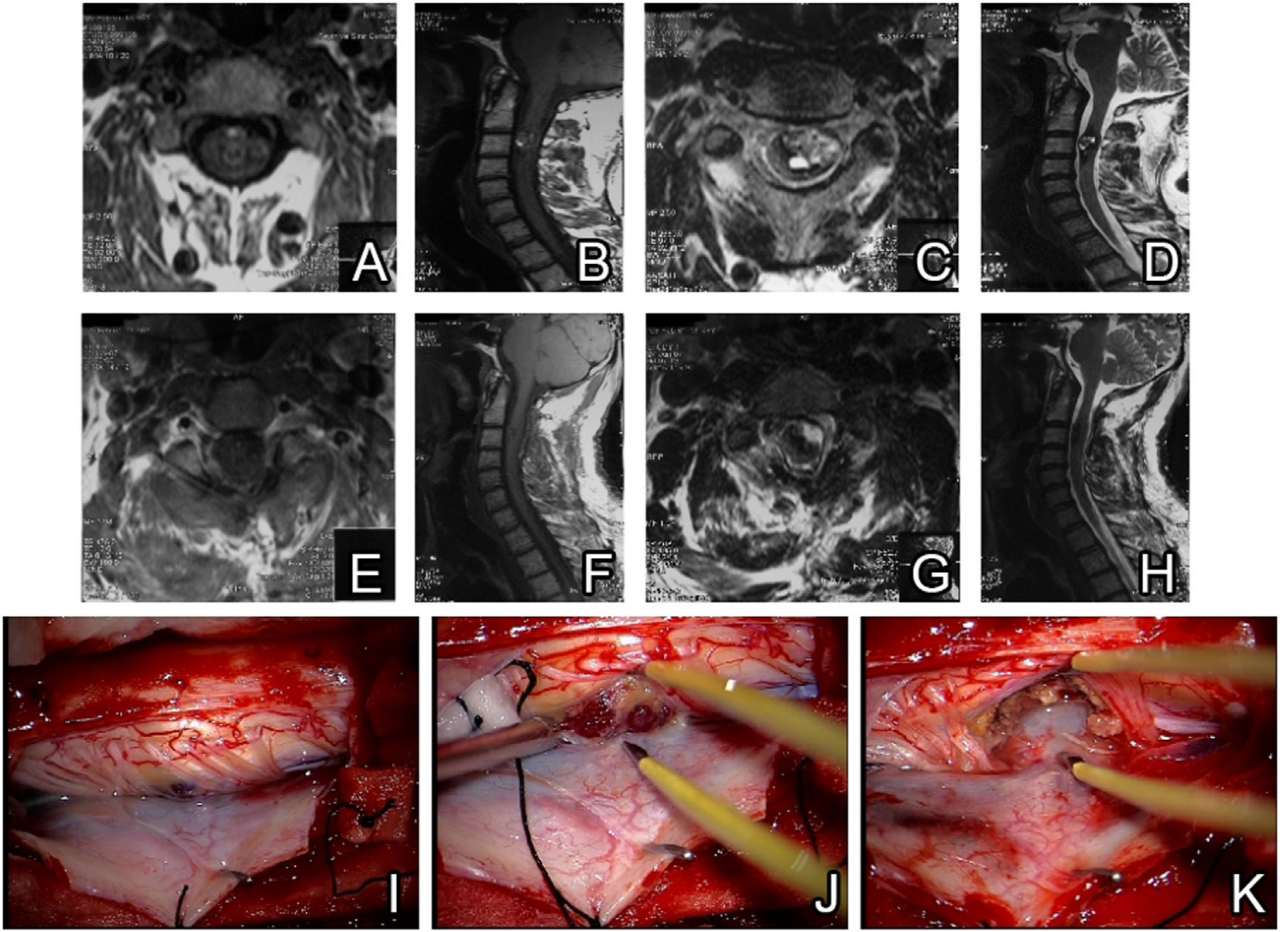
Preoperative MRI scans reveal a cervical intramedullary located cavernoma **(A–D)**. Postoperative MRI confirms total resection without hematoma or residue **(E–H)**. Intraoperative view of cavernoma after midline dural incision and retraction **(I)**. Anterior intramedullary located cavernoma seen after mild retraction of spinal cord **(J)**. After total removal of cavernoma, no residue is seen in the surgical field **(K)**.

A 46-year-old male patient with a history of neck pain for 10 years presented with loss of function in the right hand that persisted during the last month. He described bilateral numbness in both hands while sleeping at night, more pronounced in the left hand. In the neurological exam, Lhermitte sign was positive and there was a pathological Hoffman sign positivity on the left hand. Cervical MRI showed cervical intramedullary cavernoma on the C3 level. Following a C2–C3 laminectomy, lateral myelotomy was performed and the cavernoma was totally excised. A control MRI was performed postoperatively on day 1 and total excision was confirmed radiologically. With no additional complaints, the patient was discharged on day 3 of post-op (Figure 2).

#### Case-2

**Figure 3:**
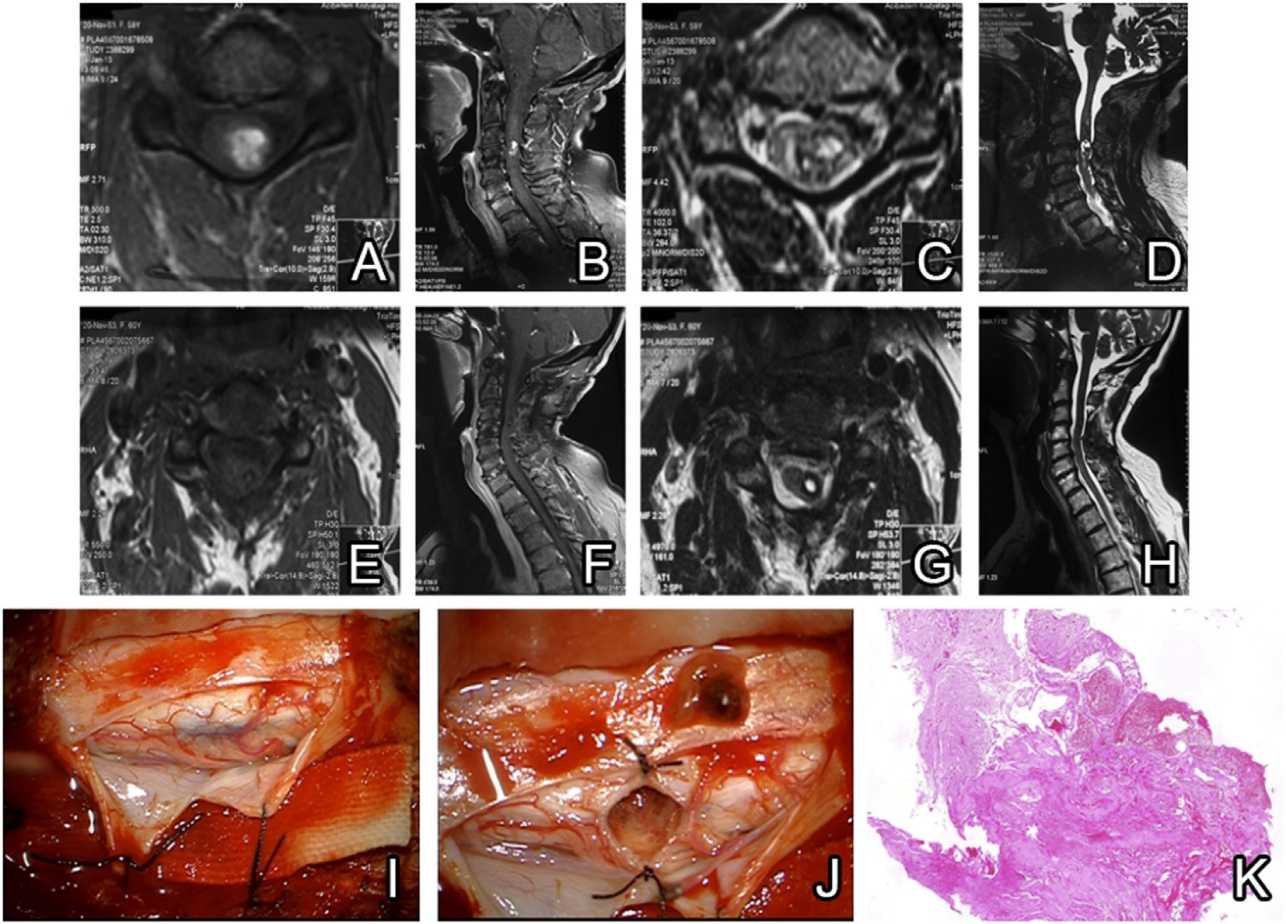
Preoperative MRI scans show a cervical cavernoma with intracavernous hemorrhage causing heterogenous appearance in sections **(A–D)**. Postoperative MRI confirms total resection without hematoma or residue **(E–H)**. Posterior intramedullary cavernoma can be seen under the duramater **(I)**. After removal of the cavernoma, total resection is confirmed intraoperatively and resected cavernoma is seen in the field **(J)**. Hematoxylin and eosin staining of hematoma tissue; compact clusters of vessels without intervening neuropil and reactive gliosis with hemosiderin-laden macrophages are observed **(K)**.

A 59-year-old female patient presented with numbness and spasm in her left hand lasting for the last month. No pathology was detected during the neurological examination. Cervical MRI showed a lesion in the C3–C4 level, which may be compatible with a cavernoma. A C3–C4 laminectomy was performed on the patient and after a left lateral myelotomy, a brown, lobulated cervical intramedullary cavernoma with cystic component was revealed. It was completely excised and the postoperative MRI on day 1 showed total removal. The patient was discharged on the day 4 of post-op with no neurological deficits (Figure 3).

#### Case-3

**Figure 4:**
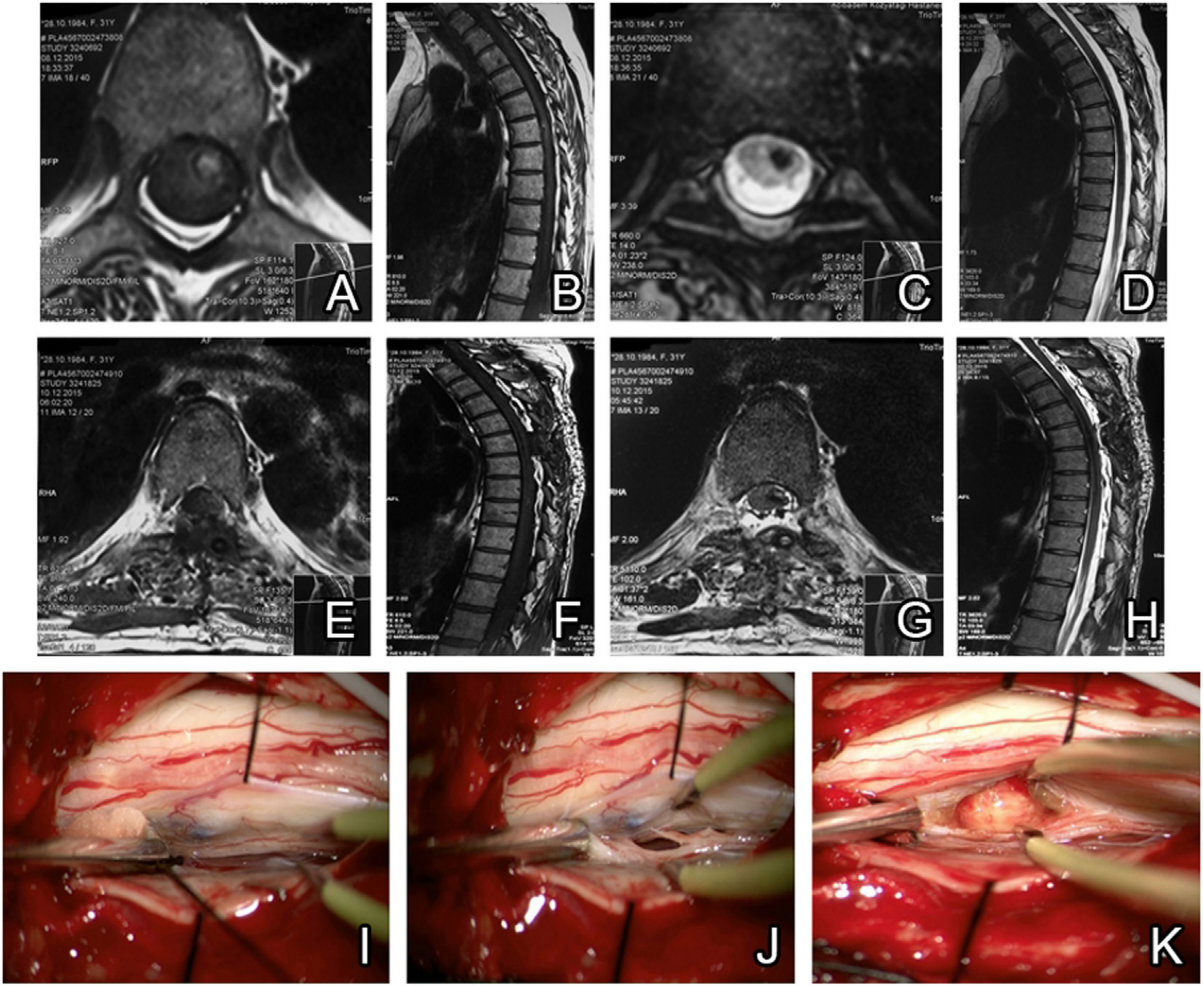
Preoperative MRI scans reveal a thoracal intramedullary located cavernoma. T1 axial and sagittal sections showed hyperintense view of cavernoma **(A,B)**, but hypointense view of cavernoma seen in T2 axial and sagittal sections **(C,D)**. Postoperative MRI confirms total resection without hematoma residual cavernoma **(E–H)**. Intraoperative view of cavernoma before resection **(I,J)**. Gross total resection is confirmed after removal **(K)**.

A 31-year-old female patient presented with back pain, which started in the last month. Loss of strength in the left leg, loss of sensation in both legs, abdomen, and the perineum was observed. In the neurological exam, the left leg had 4/5 muscle strength. Hypoesthesia was present bilaterally below T6 and both legs. Deep tendon reflexes were hyperactive bilaterally in the lower extremities. Spinal MRI showed intramedullary spinal cavernoma at the T6 level and the patient was operated. During the operation cavernoma, location was checked by intraoperative USG and neuro-monitorization was performed. Dura was opened as a result of T6 laminectomy and, by means of dorsal myelotomy, cavernoma tissue was approached and totally resected. A control MRI was performed postoperatively on day 1 and total excision was confirmed. The patient’s pain subsided and was discharged on day 4 (Figure 4).

## Discussion

A cavernoma presents itself usually as a mulberry-shaped vascular anomaly, formed by an abnormal cluster of small sinusoidal-type capillaries and venules that create lesions in the CNS. Cavernomas originate from blood vessel progenitors whose development is arrested (1, 2). They are dynamic lesions that may change size and shape over time by thrombosis, intralesional hemorrhage, cyst formation, and involution of caverns. A very typical feature of cavernomas is hemosiderin deposition, which is thought to arise from clinically silent small bleedings that create cavities around the blood vessels, subsequently leading to hemosiderin accumulation in the surrounding neural tissue. Hemosiderin accumulation is clinically relevant, since it is observed as a hypointense signal ring around the lesion in MRI (11).

Cavernoma is mostly observed between third and sixth decades of life (12). A significant portion of patients are around 40 years old; however, literature findings also report patients ranging from 2 to 80 years old individuals (13, 14). There is a clear difference among genders in prevalence, women constituting approximately 70% of all cavernoma patients; however, this pattern was not observed in our patients, incidence rate was equal for both sex (15). On the other hand, the age range in our series is in line with literature findings.

Depending on the location and growth rate of tumors, patients apply to clinics with one or more of the following symptoms and complaints: spinal pain, radiculopathy, progressive paraparesis, acute paraplegia, and progressive myelopathy (2). Progressive compressive myelopathy is the most commonly reported among those symptoms. An acute onset of symptoms is rare, most of them occur only after hemorrhage.

### Natural History of Cavernomas Is Not Well Understood

The number of prospective studies and the number of patients followed in such studies are very limited. Due to the dynamic nature of the lesions and the risk of bleeding, conservative treatment is not advised for most of the spinal cavernoma cases. Hemorrhage rates per year for cavernomas are estimated to be between 1.7 and 4.5% indicating a considerable risk factor for patients (16). To avoid the risk of bleeding, surgery upon diagnosis with the goal of total resection should be the primary strategy in treatment. Total resection is quite feasible and achieved, in 94% of all reported attempts; all of our patients add more data for the achievement of total resection (16). For the cases in which the surgery is delayed, patients are at a greater risk for neurological deterioration, presumably due to scarring around cavernoma, which renders dissection more invasive (14). Total resection should be confirmed both during the surgery and postoperatively, since cavernoma residues may cause neurological deterioration due to their tendency to grow and bleed. The first report that explained the surgical removal of cavernoma was published by Bremer and Carson in 1890 (17). Since then, advances in imaging and surgery methods facilitated the diagnosis and treatment of cavernomas considerably.

There are reports in the literature advocating for the employment of “wait and see” protocol for certain patients (18, 19). With the exception of intracerebral cavernomas, our experiences guide us to maintain that surgery has to be the main and preferred treatment protocol for cavernomas in all patient populations. It has been kept in mind that it is standard procedure for most elderly patients to perform surgery for conditions like lumber stenosis, disc herniation, and listhesis. Therefore, we believe the same group of elder patients can and should undergo surgical operation in the presence of cavernoma. Microsurgery techniques enabled gross resection surgeries with acceptable morbidity and also no reports of mortality are found in the literature. Certainly, patients who are below 60 points under Karnofsky scale and/or with serious comorbidities should be treated conservatively. However, we suggest that for the rest of the patient population, there exists no clear rationale for avoiding or postponing the surgery.

Surgery-related complications and morbidity are generally a result of ischemic damage that occurs secondarily due to sustained traction, manipulation, rotation, and overheating of the spinal cord during bipolar coagulation (20). Intraoperative monitoring is essential in predicting the degree of surgical manipulations that spinal cord can tolerate. Despite the fact that electrophysiological methods can give false negative and positive results on some occasions, electrophysiological motorization remains an important tool in avoiding surgery-related deficits. Exact preoperative localization of the lesion is another critical factor in reducing morbidity. Relying on preoperative imaging for localization may fail as the displayed anatomy of the lesion may shift during surgical approach. Intraoperative ultrasound imaging is a reliable method in helping the precise localization of the lesions. In our case series, all patients were intraoperatively monitored and intraoperative ultrasound guidance was used.

## Conclusion

In the light of our literature review and experience with patients with cavernoma, we recommend neither a wait and see protocol nor a conservative treatment. It is safe to conclude that, under optimum conditions, microsurgical operations enable favorable neurological outcome for patients. Results from our case report indicate a low morbidity rate and consistent improvements over time in neurological outcomes.

Patients must be diagnosed with MRI since it is nowadays a gold standard. Preoperative and postoperative scores are important in evaluating the patients’ condition and improvement. The results from our patient series also reinforce the notion that immediate surgery should be the preferred treatment method for cavernomas. Intraoperative neurophysiologic monitorization should assist the surgery in order to prevent complications. In conclusion, microsurgery is a gold standard method that we recommend for cases of cavernomas, which will not recur if gross total resection is achieved.

## Author Contributions

IS is the corresponding author and the first author. MP is the second author.

## Conflict of Interest Statement

The authors declare that the research was conducted in the absence of any commercial or financial relationships that could be construed as a potential conflict of interest. The reviewer MR and handling Editor declared their shared affiliation.
